# Changes in Plasma Levels of Prestin and Otolin-1 in Dental Students

**DOI:** 10.7759/cureus.75719

**Published:** 2024-12-14

**Authors:** Jeung Woon Lee, Stephan Miser, Kyle O'Connor, Carter Bedinghaus, Gabrielle Tomeo, Mary Badawy, Don Millner

**Affiliations:** 1 School of Dental Medicine, Lake Erie College of Osteopathic Medicine, Bradenton, USA

**Keywords:** dental students and medical students, inner ear, noise-induced hearing loss, otolin-1, outer hair cells, prestin

## Abstract

Introduction: Dentists and dental professionals report a high prevalence of noise-induced hearing loss (NIHL) and related symptoms. Chronic exposure to high-frequency dental instrument sounds, which can damage the outer hair cells (OHCs) of the cochlea, is strongly linked to their NIHL. Similarly, dental students in teaching clinics often report symptoms associated with NIHL. In this study, we measured plasma levels of prestin and otolin-1, two biomarkers associated with inner ear health, in first-year (D1) and third-year (D3) dental students. D1 students were selected for their relatively short exposure to dental clinics, while D3 students represented a group with substantial cumulative exposure. First-year (M1) medical students, who have no exposure to dental instruments, served as the negative controls, while dental faculty, many of whom are diagnosed with NIHL or self-report hearing problems, served as the positive controls.

Methods: Thirty-one students (D1=11, D3=8, M1=12) and 10 dental faculty volunteered for the study. Participating students completed an online survey about their hearing health, exposure to excessive sounds within and outside school, and use of hearing protection. Sound levels of medical Osteopathic Manipulative Medicine (OMM) and dental Simulation (SIM) labs while in session were recorded with a calibrated sound meter. Plasma levels of prestin and otolin-1 were quantitated using commercial ELISA kits.

Results: The sound level of in-session OMM lab was significantly higher (72.64 ± 1.69 dBA) than SIM lab (65.37 ± 3.97 dBA). Both medical and dental students experienced similar exposure to outside school noises; however, dental students had significantly longer exposure to high-frequency dental instrument sounds in the SIM lab (12.3 hours/week; 74.93-80.51 dBA). Plasma prestin levels were lowest in D3 students (559.88 ± 24.91 ng/ml), followed by D1 students (576.27 ± 71.44 ng/ml). Negative control M1 students had higher levels (675.73 ± 90.23 ng/ml), while the positive control dental faculty group exhibited the highest prestin levels (727.71 ± 128.65 ng/ml). Prestin levels in D3 students were significantly lower than those in M1 students, and the dental faculty group had significantly higher prestin levels than both D1 and D3 students. No differences in otolin-1 levels were observed among the groups.

Conclusions: Our finding that D3 students, with greater experience using dental instruments in the SIM lab, exhibited the lowest plasma prestin levels may indicate a protective mechanism by the OHCs, downregulating its expression to reduce the risk of NIHL. This is in line with the findings in the dental faculty, who self-reported NIHL or hearing problems and exhibited the highest prestin levels. The lack of changes in plasma otolin-1 levels across groups, combined with students self-reporting excellent hearing health, may offer alternate view of subclinical inner ear damage among dental students. Future studies incorporating audiometry and otoacoustic emission tests along with the inner ear biomarkers may provide better understanding of NIHL in dental students.

## Introduction

Occupational hearing loss may come about through various mechanisms, such as physical trauma to the ear canals or damage to the middle ear structures, but chronic exposure to high-frequency vibrations and high-level sounds may be a leading cause [1-3]. Industrial workers and military personnel exposed to chronic high frequency sounds from operational equipment report high prevalence of noise-induced hearing loss (NIHL) ranging from 9% to 67% [4,5]. Similarly, the Centers for Disease Control and Prevention estimated prevalence of NIHL among teenagers between 13% to 17.5% from exposure to excessive noise [6,7]. The National Institute on Deafness and Other Communication Disorders describes chronic and repeated exposure to sound levels at or exceeding 85 dBA may lead to NIHL, and the Occupational Safety and Health Administration indicates implementation of hearing conservation program when a worker’s sound exposure exceed an 8-h time-weighted average as a preventive measure for NIHL [8].

Among health professionals, dentists and dental practitioners have been described as having one of the highest incidences of NIHL, primarily associated with their prolonged exposure to high-frequency noises from dental equipment, such as high-speed handpieces and ultrasonic scalers [9-11]. The incidence and severity of NIHL among dentists seems to correlate with their length of practice, with selective damage primarily to the left ear [8]. 

Several studies also report that dental students in teaching clinics in dental school are regularly exposed to sound levels ranging from 65 dBA to 79 dBA, with peak levels reaching 89 dBA and 99 dBA [12-14]. Additionally, many dental students report having clinical signs of NIHL, including frequent tinnitus, noise-annoyance, and reduced hearing thresholds at 4 kHz-6kHz in the left ear [15]. Animal and clinical studies report NIHL, and sensorineural hearing loss (SNHL) are strongly associated with damage of the outer hair cells (OHCs) in the organ of Corti within the cochlea [16-19]. Damaged or dying OHCs release their cytoplasmic content into blood, and the serum detection of these proteins have been used as biomarkers for health of OHCs [20,21]. To date, there have been no studies that quantitatively examined these cellular biomarkers associated with NIHL, SNHL or OHC injury in dental students or dental professionals.

Prestin and otolin-1 are two promising cellular biomarkers associated with inner ear injury [22,23]. Prestin, a motor protein exclusive to the cochlear OHCs, facilitates their electromotility and plays an essential role in sound amplification and frequency selectivity in the cochlea. Noise-induced cochlear injuries can cause prestin to be released into the perilymph, diffuse into CSF, and subsequently entering bloodstream [23]. Serum prestin levels may correlate with the severity of hearing loss, and recent studies have reported detection of prestin in individuals with NIHL/SNHL, reinforcing its potential as a biomarker for early detection of hearing loss. Otolin-1, another protein associated with the inner ear, is primarily involved in the vestibular system but is also found in the cochlea. It plays a key role in the formation of otoconia, essential structures for balance and spatial orientation within the vestibular system. Elevated levels of otolin-1 in serum have been associated with noise-induced inner ear damage or ototoxicity [22,24].

In the present study, we investigated whether dental students exhibited damage to OHCs and inner ear structures by quantitating the plasma concentrations of prestin and otolin-1. We examined the blood samples from healthy first-year (D1) and third-year (D3) dental students during the final week of their respective academic years. These two groups were selected to represent dental students with shorter (D1) and longer (D3) durations of exposure to high-frequency dental instrument sounds in dental school.

## Materials and methods

Participants

This study is part of a larger experiment that examines the effects of chronic exposure to dental instrument sounds on dental students and the subsequent cellular changes in the inner ear structures. For this study, 19 dental students (D1, n=11; D3, n=8), 12 medical students (M1), and 10 dental faculty participated. The M1 students, who were not exposed to dental instrument sounds, served as the age-matched negative control group. The dental faculty, the majority of whom are either diagnosed with hearing problems or self-reported having hearing problems, served as the positive control group.

The participation exclusion criteria for all dental/medical students were: individuals diagnosed with hearing loss and/or vestibular problems and usage of hypertensive medication. Before enrollment, all participants received information about the research protocol and voluntarily completed the Informed Consent form. The study was reviewed and approved by the LECOM Institutional Review Board (Protocol Number: 31-028) in accordance with US federal regulations for human research, and the 1964 Helsinki Declaration.

Online survey

All student participants (n=31) were asked to complete an online questionnaire that gathered demographic information, individual perception of hearing changes, exposure to excessive sounds within-school and outside-school environments, presence of tinnitus, and usage of hearing protection tools during the past 12 months.

Sound level measurement and exposure duration

Sound levels (dBA) were measured using a calibrated sound meter (XRC Electronics, Shenzhen, China) to assess both the level and duration of sound exposure by the medical and dental students in three distinct school environments: the Osteopathic Manipulative Medicine (OMM) medical students' lab, the Simulation (SIM) dental students' lab, and the library. Measurements were conducted on four randomly selected days, with sound levels recorded at 10 randomly chosen locations within each environment per day. Additionally, sound levels generated by the dental electric drill, air/water syringe, and ultrasonic scaler in the SIM lab were measured while operated by the dental students. These instruments were chosen as they were most widely used by the dental students during the SIM lab sessions. For these measurements, the sound meter was positioned next to the student's ear to estimate the sound levels perceived by the ears. Lastly, the duration of student exposure to these instrument sounds (hr/wk) and time spent in OMM or SIM labs (hr/wk) were recorded to evaluate the cumulative weekly sound exposure. The hours of sound exposure outside of class (independent practice) time were not included.

Quantitation of plasma prestin and otolin-1

Each participant provided a blood sample from a fingertip using a spring-loaded lancet. Approximately 7 to 9 drops of blood were collected into a BD Vacutainer EDTA (purple top, ThermoFisher Scientific, Waltham, MA) tube. All blood samples were collected between 3 pm and 5 pm during the last academic week of the spring semester. Upon collection, the blood samples were briefly stored in a cooler at 4°C, and then centrifuged at 2500 G for 10 minutes. The plasma samples were transferred to clean Eppendorf tubes and stored at -80°C until processed for the prestin and otolin-1 biomarker assays. The plasma concentration of prestin and otolin-1 were quantitated using the commercially available ELISA kits (MyBioSource Inc., San Diego, CA), following the procedures described by the manufacturer. Each plasma sample was tested in duplicate, and the average of the two values was used for the analysis.

Statistics

The plasma data were assessed for normality and variance homogeneity using the Shapiro-Wilk and Levene’s tests, confirming a normal distribution. Differences among groups were analyzed using a one-way ANOVA, followed by Tukey’s post-hoc test. Prestin and otolin-1 data are presented as means with standard deviations. For the online survey data, the categorical data were analyzed using the Kruskal-Wallis test, and the exposure to excessive sound data was analyzed using the Mann-Whitney U test for comparison between groups. For the sound level (dBA) data, the differences among the conditions were analyzed using the Kruskal-Wallis test followed by the Dwass-Steel-Critchlow-Fligner pairwise comparisons posthoc test. Statistical analyses were performed using GraphPad Prism v10.4 (Dotmatics, Boston, MA) and jamovi v2.38 (https://www.jamovi.org), with p-values <0.05 considered statistically significant.

## Results

Online survey analysis

Of the 31 student participants, 27 completed the online survey (Table 1). There were no statistical differences between the subgroups for age, hearing health (scale of 1 (bad) to 5 (excellent)), and frequency for tinnitus. All students self-reported their hearing health as excellent or near-excellent with an average score of 4.5 or greater. Exposure to excessive sound from outside school (hr/wk), such as traffic, construction, music, and hobby-related noises, showed no statistical difference between dental and medical students. Dental students, however, reported significantly higher in-school exposure to sounds from dental electric drills, air/water syringes, and ultrasonic scalers, averaging 12.3 hours per week during class time. Medical M1 students reported no exposure to such sounds within the school environment (Table 1).

**Table 1:** Survey results on participant hearing health and exposure to excessive sounds within and outside school environments. #: Excessive Sound: Any sound that is consciously bothersome to the health and well-being. *: Statistically significant between the student groups (Mann-Whitney U test, p<0.05). All student participants self-reported as having excellent to near-excellent hearing health (≥4.5; scale 1 to 5). Both dental and medical students were exposed to similar types and duration of excessive sounds from outside school sources. In addition, only the dental students reported exposure to high frequency dental instrument sounds ranging from 2.3 hr/wk (ultrasonic scaler) to 12.3 hr/wk (dental electric drill) from SIM lab.

Variables	Dental Students		Medical Students	
	Female	Male	Female	Male
Participants	5	10	5	7
Age	25.2	24	25.2	25.7
Hearing health [1 (Bad) to 5 (Excellent)]	4.5	4.7	5	4.6
Tinnitus severity (Frequency/day)	1 (3/day)	1 (1/day)	0	1 (1/day)
Exposure to excessive sound# (Hr/Wk)				
Inside school				
Dental electric drill	12.3*		0	
Dental air/water syringe	11.3*		0	
Ultrasonic scaler	2.3*		0	
Loudspeaker (PA system)	0.4		0.1	
Outside school				
Auto/traffic noise	2.3		3.9	
Construction noise	0.1		0.6	
Concert/party noise	1.1		1.3	
Hobbies (boating, car, Jet-ski)	0.8		0.7	
Ear protection (in school)				
Yes	40%		0%	
No	60%		100%	
Types of ear protection				
Ear plugs (foam/silicone)	34%		0%	
Earphones with ANC	66%		0%	

Sound level measurement and exposure duration

Using a calibrated sound meter, the sound levels (dBA) were measured at three different academic locations (library, OMM lab, and SIM lab) and from three dental instruments (dental electric drill, air/water syringe, and ultrasonic scaler; Table 2). The library had the lowest sound level at 39.16±0.11 dBA, followed by the SIM lab and OMM lab at 65.37±3.97 dBA and 72.64±1.69 dBA, respectively. The Kruskal-Wallis test showed that the sound levels of both labs were significantly higher than the library (p<0.001), and the sound level of the OMM lab was significantly higher than the SIM lab (p<0.001). Dental students also spent significantly more time in the SIM lab using dental instruments compared to the time medical students spent in the OMM lab (p < 0.001). Each of the hand-held dental instruments produced sound levels that were significantly higher than those observed in both the OMM and SIM labs while in session (Table 2, p<0.001). 

**Table 2:** Quantitation of sound levels (dBA) from school environments associated with the medical and dental students. *: Indicates only the academic session hours; hours outside the academic session are not included. #: Statistically different vs. OMM Lab and Library (p<0.001). &: Statistically different vs. SIM Lab (p<0.001). The sound levels in the OMM lab during active sessions were significantly higher than those in the SIM lab during its active session. Overall, dental students spent significantly more hours/week in the SIM lab than medical students in OMM lab. Additionally, dental students were exposed to dental instrument sounds, which had significantly higher sound levels than those in the active session OMM lab (Kruskal-Wallis test with Dwass-Steel-Critchlow-Fligner pairwise post hoc test).

Variables	Sound Level (dBA ± SD)	Exposure* (hr/wk)
Environment		
Library	39.16±0.11	
OMM Lab (medical)	72.64±1.69^#&^	2^&^
SIM lab (dental)	65.37±3.97^#^	6
Instruments		
Dental electric drill	74.93±1.44^#^	5
Air water syringe	78.33±4.62^#^	0.5
Ultrasonic scaler	80.51±2.23^#^	1

Quantitation of plasma prestin and otolin-1

**Figure 1:**
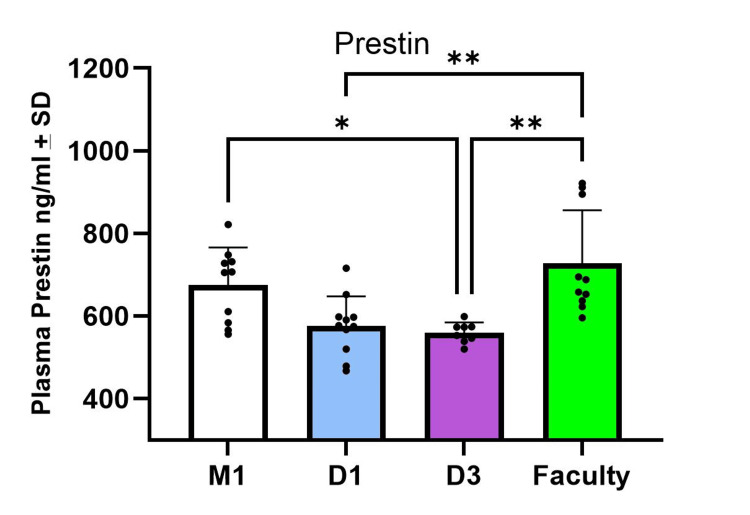
Plasma prestin concentrations in the study groups. One way ANOVA showed that the plasma prestin levels of D3 students were significantly lower than both negative control M1 students and positive control dental faculty group. The D1 dental students also had significantly lower prestin levels than dental faculty group. *: p<0.05; **: p<0.005

The plasma prestin level for M1 students (negative control) was 675.73±90.23 ng/ml, while the dental faculty group (positive control) had 727.71±128.65 ng/ml. For the dental students, D1 and D3 groups had levels of 576.27±71.44 ng/ml and 559.88±24.91 ng/ml, respectively (Figure 1). 

A one-way ANOVA showed significant differences between the groups (F(3, 35) = 7.79, p<0.001). Tukey’s post hoc analysis showed that the D3 students had significantly lower plasma prestin levels compared to the M1 students (p<0.05). Additionally, the dental faculty group had significantly higher prestin levels than both D1 and D3 students (p<0.005).

**Figure 2:**
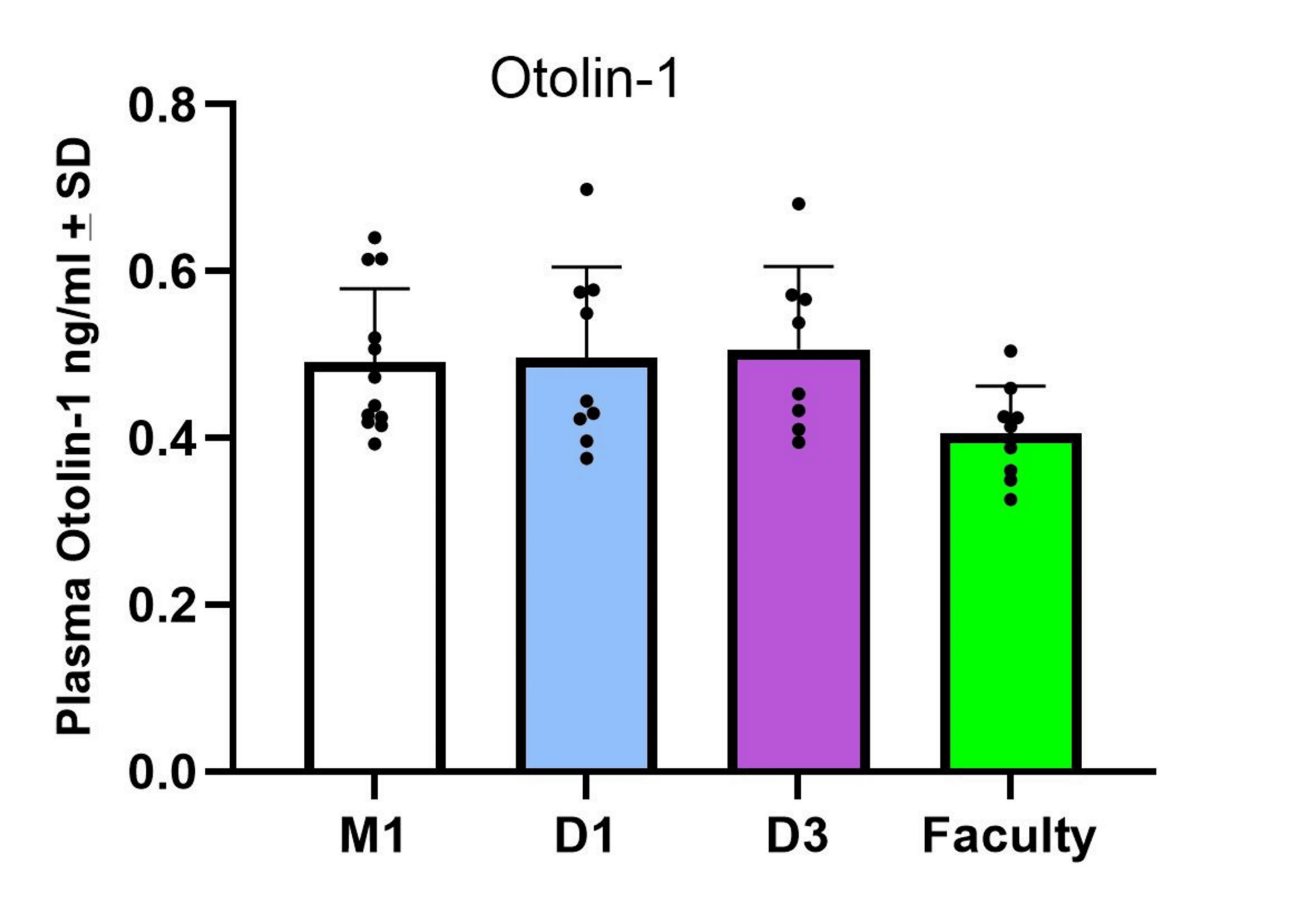
Plasma otolin-1 concentrations in the study groups. The plasma otolin-1 levels were not different among the groups examined.

For the plasma levels of otolin-1, the M1 students and dental faculty groups had 0.48±0.09 ng/ml and 0.41±0.06 ng/ml, respectively. The D1 and D3 students had plasma otolin-1 levels of 0.50±0.11 ng/ml and 0.51±0.10 ng/ml, respectively (Figure 2). 

A one-way ANOVA showed there was no statistical difference among the groups (F(3,35) = 2.39, p=0.09).

## Discussion

This study examined the plasma concentration of prestin, a potential biomarker for outer hair cell (OHC) function and noise-induced hearing loss (NIHL)/sensorineural hearing loss (SNHL), and otolin-1, a biomarker for vestibular dysfunction, in first-year (D1) and third-year (D3) dental students exposed to high-frequency dental instrument sounds during their training in dental school. The first-year (M1) medical students, who do not have exposure to dental instruments, and dental faculty, who already self-report having hearing problems, participated as the negative and positive control groups, respectively. Data from our study showed three important findings. First, both D1 and D3 students had lower plasma prestin concentrations compared to the negative control M1 student group, with D3 students showing significantly lower prestin levels than M1 students. Second, the dental faculty group had significantly higher concentrations of prestin than D1 and D3 students, as well as the control M1 students, though the difference with the latter was not statistically significant. Third, plasma otolin-1 concentrations in D1 and D3 students remained unaffected and were comparable to those of M1 students.

Prestin is a motor protein found in the OHCs of the cochlea, essential for their electromotility, and amplifies sound signals to improve auditory sensitivity. Damage to OHCs, a characteristic feature of NIHL/SNHL, leads to the release and sequential distribution of prestin into perilymph, CSF, and blood, making it a potential biomarker for cochlear injury [23]. Our data showed that in healthy dental students (mean age = 24.89), the chronic exposure to dental instrument sounds in the SIM lab was associated with having lower plasma prestin concentrations in both D1 and D3 students compared to negative control M1 students. The decrease was statistically significant only with the D3 students who had more years of instrument sound exposure. Even though the M1 students in the OMM lab were exposed to significantly higher sound levels than dental students in the SIM lab, it was the latter group that had much lower prestin concentrations indicating such reduction may be associated with the length of the exposure to chronic dental instrument sounds as compared to the loudness of sound level itself. 

Our results are in agreement with previous reports that showed a decrease in plasma prestin levels in healthy individuals exposed to high-frequency sounds or those experiencing noise-induced hearing loss and/or ototoxicity [22,24,25]. Parker et.al. (2022) reported that in healthy young individuals (mean age = 20.26), the At-Risk group (daily noise doses exceeding 100% of 3wk average; average daily exposure levels = 85.95 dBA) showed significantly reduced levels of serum prestin compared to the Low-Risk group [25]. Minoretti et.al. (2024) also reported that in helicopter pilots (mean age = 39.1) and construction workers (mean age = 38.9), who are regularly exposed to loud occupational sounds, their serum prestin concentrations were significantly lower compared to controls (office workers; mean age = 38.7) [24]. All participants had reported normal auditory functions at the onset of the study.

The reduction in plasma prestin concentrations seen in D1 and D3 students may be associated with the environmental downregulation of prestin expression by the OHCs, as proposed by Parker et.al. [25]. The authors described that in healthy young individuals, when experiencing continuous loud high-frequency sounds, the OHCs may not need to maintain a high cochlear amplification function, thereby down-regulating the prestin expression to compensate for possible cochlear damage [26]. In our study, the D3 dental students, who self-reported having excellent hearing health and had more years of exposure to high-frequency dental instrument sounds, had the lowest plasma prestin levels. Such initial protective physiological events, when challenged with continuous high-frequency sound levels, may lead to damage to OHCs leading to clinical conditions associated with NIHL/SNHL. The rise in plasma prestin concentrations in individuals with SNHL is well documented [21,26,27], and also observed in our study. The dental faculty group, who self-reported as having hearing problems, had the highest average plasma prestin levels of all student groups. Sun et al. (2020) reported that individuals with idiopathic sudden sensorineural hearing loss (ISSHL, mean age = 57.9) had significantly higher plasma prestin levels compared to age-matched controls [28]. Similar results were reported by Saadat et.al. (2021) with ISSHL patients (mean age = 47.8) having significantly higher plasma concentrations of prestin to age-matched control [19]. Therefore, the high plasma prestin levels observed in our study by the dental faculty may be strongly associated with damage to OHCs.

Plasma otolin-1 levels in dental students were unaffected by high-frequency dental instrument sounds and were comparable to those of medical students. While changes in prestin levels may not always be associated with changes in otolin-1 levels, Minoretti et. al. (2024) reported that helicopter pilots with normal auditory function showed decreased prestin and increased otolin-1 levels [24]. Similarly, Solis-Angeles et al. (2021) observed comparable changes in serum prestin and otolin-1 levels in pottery workers with varying blood lead concentrations [22]. The unchanged otolin-1 levels in dental students may support the view that chronic exposure to high-frequency dental instrument sounds may not be sufficient to lead to subclinical inner ear damage, as observed in helicopter pilots and pottery workers. Instead, the combination of unchanged otolin-1 levels and reduced plasma prestin may reflect an environmental downregulation of prestin expression as a protective response to prolonged exposure to high-frequency instrument sounds, rather than subclinical injury to the OHCs. More tests, however, would be needed for the confirmation of environmental adaptation and downregulation hypothesis of prestin.

 There are limitations to our study that should be considered. The participant sample size is relatively small compared to other studies that investigated blood prestin levels. A larger sample size may provide greater statistical power to detect significant differences in plasma prestin levels between D1 and M1 students. A follow-up study is currently underway to address this limitation. Hearing assessment tests were not included in this study; instead, a self-reported online survey was used as a measure of participants' hearing health. This approach makes it challenging to establish a direct relationship between changes in plasma prestin levels and potential subclinical OHC damage. Future studies incorporating audiometry and otoacoustic emission tests along with quantitation of inner ear biomarkers may provide additional insight into the condition of OHCs and the occurrence of NIHL/SNHL in dental students exposed to high-frequency dental instrument sounds.

## Conclusions

This study is the first to evaluate prestin and otolin-1 as potential biomarkers for hearing changes in dental students during their training in dental school. Our results showed that prestin levels decreased in dental students exposed to high-frequency dental instrument sounds, with longer exposure associated with a greater decrease in prestin. In contrast, otolin-1, a biomarker for vestibular dysfunction, remained unaffected. These results suggest that exposure to high-frequency dental instrument sounds may not be sufficient to cause OHC-related cellular damage. Instead, the observed reduction in prestin levels likely reflects a protective mechanism by the OHCs, reducing prestin expression to safeguard against potential injury. Additional larger-scale cross-sectional studies would be needed to ascertain the protective physiological changes by the OHCs in dental students.

## Appendices

Individual hearing assessment - IHA questionnaire (distributed through SurveyMonkey)

This questionnaire is part of the research on Detection of Changes in Hearing Abilities in LECOM students and faculty. For your privacy protection, please use your Participant Identification number.

Excessive Sound = Any sound that is consciously bothersome to your health and well-being.

1. Please enter your Participant Identification number.  ______________________

2. Please select your biological sex?

  Male

  Female

3. Please enter your age.

  _______ years old

4. Please select which study group you belong to.

  Dental D1 student

  Dental D3 student

  Medical M1 student

Faculty

5. How would you describe the healthiness of your sound/hearing abilities at present.

  5 - healthy (normal)

  4 - slightly bad

  3 - moderately bad

  2 - bad

  1 - very bad

6. In the past 12 months (since last year), did you notice changes in your hearing abilities?

  My hearing abilities did not change during past 12 months

  My hearing abilities decreased by ______ % (subjective, write a numeric value) during past 12 months

  My hearing abilities increased by ______ % (subjective, write a numeric value) during past 12 months

7. Do you currently experience tinnitus.

  No I do not experience tinnitus

  Yes I experience tinnitus ______ (write a number) times per day

8. How would you rate the severity of your tinnitus.

  0 - none

  3 - moderate

  5 - severe

9. In the past 12 months (since last year), did you notice changes in your tinnitus?

  My frequency for tinnitus did not change during past 12 months

  My frequency for tinnitus decreased by ______ % (subjective, write a numeric value) during past 12 months

  My frequency for tinnitus increased by ______ % (subjective, write a numeric value) during past 12 months

10. Please respond to the statement: I feel that my hearing ability decreased since I started attending LECOM dental school (or your corresponding LECOM school).

  0 - Do not agree

  3 - Somewhat agree

  5 - Fully agree

11. Please respond to the statement: The frequency and severity of my tinnitus increased since I started attending dental school (or your corresponding school).

  0 - Do not agree

  3 - Somewhat agree

  5 - Fully agree

12. Describe the types of uncomfortable excessive sounds you are exposed to from your regular surroundings WITHIN the LECOM building. Identify ALL that applies. Also indicate how many hours you are exposed to it. (Excessive Sound = Any sound that is consciously bothersome to your health and well-being.)

  Dental drills

  Dental vacuum/suctions

  Ultrasonic scaler

  Loudspeakers (like fire alarm, PA system)

  Others __________________

  _______  hrs a day

  _______ hrs a week

13. Describe the types of uncomfortable excessive sounds you are exposed to from your regular life OUTSIDE of LECOM building. Identify ALL that applies. Also indicate how many hours you are exposed to it. (Excessive Sound = Any sound that is consciously bothersome to your health and well-being.)

  Auto/traffic noise

  Construction noise

  Concert/Party noise

  Hobbies (like boating, car repair, jet ski)

  Others _________

  _______  hrs a day

  _______ hrs a week

14. Do you use Ear Protection products during your dental work within the LECOM building?

  No I do not perform dental works

  Yes I use them _______ % (write a numeric value) of times during dental works/procedures

  No I do not use them during dental works/procedures

15. What type of Ear Protection equipment do you use? Select ALL that applies.

  I do not use Ear Protection equipment

  Ear plugs (foam/silicone)

  Earmuffs

  Sound cancelling headphones

  Others  ____________
